# High-Performance IGZO Nanowire-Based Field-Effect Transistors with Random-Network Channels by Electrospun PVP Nanofiber Template Transfer

**DOI:** 10.3390/polym14030651

**Published:** 2022-02-08

**Authors:** Ki-Woong Park, Won-Ju Cho

**Affiliations:** Department of Electronic Materials Engineering, Kwangwoon University, Gwangun-ro 20, Nowon-gu, Seoul 139-701, Korea; wwoong97@naver.com

**Keywords:** electrospinning, indium–gallium–zinc oxide, random-network nanowire, field-effect transistors, polyvinylpyrrolidone

## Abstract

A random network of indium–gallium–zinc oxide (IGZO) nanowires was fabricated by electrospun-polyvinylpyrrolidone (PVP)-nanofiber template transfer. Conventional electrospun nanofibers have been extensively studied owing to their flexibility and inherently high surface-to-volume ratio. However, solution-based IGZO nanofibers have critical issues such as poor electrical properties, reliability, and uniformity. Furthermore, high-temperature calcination, which is essential for vaporizing the polymer matrix, hinders their applications for flexible electronics. Therefore, sputter-based IGZO nanowires were obtained in this study using electrospun PVP nanofibers as an etching mask to overcome the limitations of conventional electrospun IGZO nanofibers. Field-effect transistors (FETs) were fabricated using two types of channels, that is, the nanofiber template-transferred IGZO nanowires and electrospun IGZO nanofibers. A comparison of the transmittance, adhesion, electrical properties, reliability, and uniformity of these two channels in operation revealed that the nanofiber template-transferred IGZO nanowire FETs demonstrated higher transmittance, stronger substrate adhesion, superior electrical performance, and operational reliability and uniformity compared to the electrospun IGZO nanofiber FETs. The proposed IGZO nanowires fabricated by PVP nanofiber template transfer are expected to be a promising channel structure that overcomes the limitations of conventional electrospun IGZO nanofibers.

## 1. Introduction

One-dimensional channel structures, such as nanofibers (NFs), nanowires (NWs), and nanorods, have been actively studied for developing advanced semiconductors; however, they encounter certain physical and technological limitations [1,2]. Owing to their unique properties such as a large surface-to-volume ratio, high transparency, and high flexibility, semiconductor NWs are considered promising building blocks of electronic, photonic, and biochemical sensors [3,4,5,6]. In particular, the NW structure prevents the extension of cracks in channels, indicating its suitability for fabricating flexible devices [7,8]. Methods used for NW formation include X-ray lithography, deep UV photolithography, vapor–liquid–solid growth, layer-by-layer self-assembly, and scanning probe lithography; however, these techniques can encounter issues owing to their complexity or expensiveness [9,10,11,12,13]. As an effective method for creating NW-like structures, the electrospinning technology enables the facile fabrication of randomly networked NFs and provides various advantages such as low cost, process simplicity, and large-area manufacturing [14,15]. However, electrospun NFs have issues related to poor electrical properties and reliability owing to the high impurity content induced by the solution-based process [16]. Furthermore, high-temperature calcination, which is essential for removing the polymer matrix in NFs, makes them unviable for practical flexible electronics applications using thermally vulnerable flexible substrates [16,17,18].

In this study, nanofiber template-transferred randomly networked indium–gallium–zinc oxide (IGZO) NWs are proposed to replace conventional electrospun NF channels. We fabricated two types of nanochannels: 1) directly electrospun IGZO NFs and 2) PVP NF template-transferred IGZO NWs. The random network pattern of an NF template was transferred to a sputter-based IGZO thin film through a wet chemical etching process, resulting in the formation of uniform IGZO NWs with a monolayer structure. Among the numerous candidate NW channel materials, IGZO exhibits excellent electrical and optical properties at low processing temperatures [19,20]. Electrospun polyvinylpyrrolidone (PVP) nanofibers were applied as an etching mask to create patterned nanochannels for field-effect transistors (FETs). Consequently, the PVP NF template transfer method developed in this study lowered the processing temperature, enabling the formation of IGZO NWs on various substrate materials. The morphology of the channel and the optical and mechanical properties of the fabricated NF-template-transferred IGZO NWs were investigated and compared with those of conventional electrospun IGZO NFs. In addition, FET devices were fabricated using the IGZO NWs and NFs as active channels, and their electrical characteristics and long-term reliability were compared.

## 2. Experimental

### 2.1. Preparation of the IGZO Precursor Solution and Electrospinning of IGZO Nanofibers (NFs)

A PVP precursor solution was prepared by stirring and dissolving PVP powder (100 mg; M_w_ ≈ 1,300,000, Sigma-Aldrich, Saint Louis, MO, USA) in ethanol (1.5 mL; ≥99.7%) at room temperature. An IGZO precursor solution was prepared by dissolving indium nitrate hydrate [In(NO_3_)_3_·*x*H_2_O], gallium nitrate hydrate [Ga(NO_3_)_3_·*x*H_2_O], and zinc acetate hydrate [Zn(CH_3_COO)_2_·2H_2_O] at a molar ratio of 2:1:1 in DMF (0.5 mL) and stirring at room temperature. Subsequently, an IGZO/PVP precursor solution was prepared by adding the dissolved IGZO solution to the PVP precursor solution. The PVP or IGZO/PVP precursor solution was loaded into a syringe with a metal pinhead, which was fixed to a syringe pump (NE-1000, New Era Pump Systems Inc., Farmingdale, NY, USA). The internal diameter of the pinhead needle was 0.635 mm. A copper plate, which was used as a grounded current collector, was placed at a distance of 20 cm from the metal pinhead, and the target substrate was fixed to the copper plate. The electrospinning was carried out for 2 min under a positive voltage of 20 kV applied between the metal pinhead and the copper plate. During NF electrospinning, the flow of the precursor solution was maintained at 0.4 mL/h, and temperature and relative humidity were maintained at 20 °C and 20%, respectively. Calcination was performed through furnace annealing at 600 °C for 30 min (with a lamp-up time of 30 min) in an O_2_ ambient atmosphere to remove the solvent and impurities from the as-spun IGZO NFs.

### 2.2. Formation of IGZO Nanowires (NWs)

**Figure 1:**
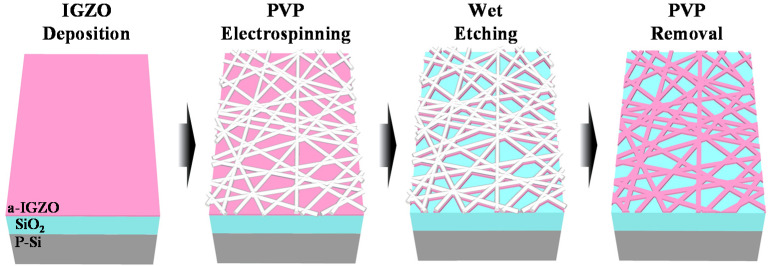
Fabrication of indium–gallium–zinc oxide nanowires (IGZO NWs) by polyvinylpyrrolidone nanofiber (PVP NF) template transfer.

SiO_2_/Si substrates were employed to form the PVP NF template-transfer-type IGZO NWs. A 20 nm-thick IGZO film was deposited using a radiofrequency (RF) magnetron sputtering system with an IGZO target (In_2_O_3_/Ga_2_O_3_/ZnO = 4:2:4.1 mol.%). The sputtering was performed at RF power of 100 W, working pressure of 6.0 mTorr, and Ar flow rate of 30 sccm. Then, PVP NFs were electrospun on the IGZO layer and baked at 200 °C for 10 min on a hot plate in air to serve as an etching mask. Subsequently, the IGZO layer was wet-etched in a 30:1 buffered oxide etchant (BOE) using the cured PVP NFs as an etch mask. Consequently, the pattern of the electrospun PVP NF template was transferred onto the IGZO film to form a random network of IGZO nanowires. Finally, the PVP NF template was removed by O_2_ plasma treatment using a reactive ion etching (RIE) system. Figure 1 illustrates the formation of the IGZO NWs by PVP NF template transfer. The electrospun PVP NFs were formed only a few layers and had large open areas. IGZO exposed by these large open areas was etched in the wet etch process. The diameter of the PVP NFs used as the etch mask was approximately 500 nm, which was much larger than the thickness of the IGZO layer. Accordingly, the loss in the horizontal direction in the wet etch process was almost neglected.

### 2.3. Fabrication of IGZO NF- and IGZO NW-Based FETs

Staggered bottom-gate type FETs were fabricated using the electrospun IGZO NFs and PVP NF template-transfer-type NWs as active channels. A p-type bulk Si (100) substrate with a 100 nm-thick thermally grown SiO_2_ layer was cleaned using a standard Radio Corporation of America (RCA) cleaning protocol. The IGZO NFs or NWs were formed on the Si/SiO_2_ substrates, followed by photolithography and wet etching in 30:1 BOE to define the active regions of the FETs. To eliminate defects and improve the electrical properties of the FETs, the IGZO NF channel was heated at 600 °C for 30 min in an O_2_ ambient, whereas the IGZO NW channel was heated at 250 °C for 30 min in N_2_ ambient. Subsequently, source and drain (S/D) electrodes were formed by depositing a 150 nm-thick Ti film using an electron beam evaporator, followed by a lift-off process. The channel length and width of the fabricated FETs were 100 μm and 80 μm, respectively.

**Figure 2:**
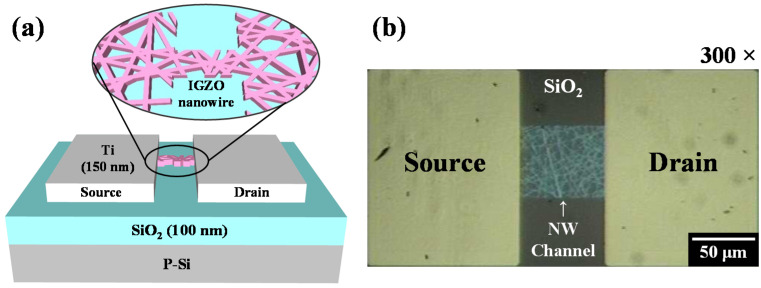
(**a**) Schematic and (**b**) optical microscopy image (300×) of the IGZO NW-based field-effect transistor (FET) fabricated by PVP NF template transfer.

Figure 2 shows a schematic and an optical microscopy image (300× magnification) of the fabricated IGZO NW FETs, which corroborated the formation of the NW pattern in the channel region of the FET.

### 2.4. Characterization

Optical microscopy images of the fabricated IGZO NFs, IGZO NWs, and FETs were acquired using a high-magnification optical microscope (SV-55, SOMETECH, Seoul, Korea). The optical transmittances of the IGZO NWs and NFs were measured using an Agilent 8453 UV–vis spectrophotometer (Hewlett-Packard Co., Palo Alto, CA, USA) in the wavelength range of 190–1100 nm. The surface morphologies of the IGZO NWs and NFs were examined using a DektakXT Bruker stylus profiler (Bruker Corp., Billerica, MA, USA). The electrical characteristics of the fabricated IGZO NW- and IGZO NF-channel FETs were evaluated using an Agilent 4156B precision semiconductor parameter analyzer (Agilent Technologies, Wilmington, DE, USA). The measuring devices were placed in a dark box during the electrical measurements to prevent external electrical and optical noise.

## 3. Results and Discussion

**Figure 3:**
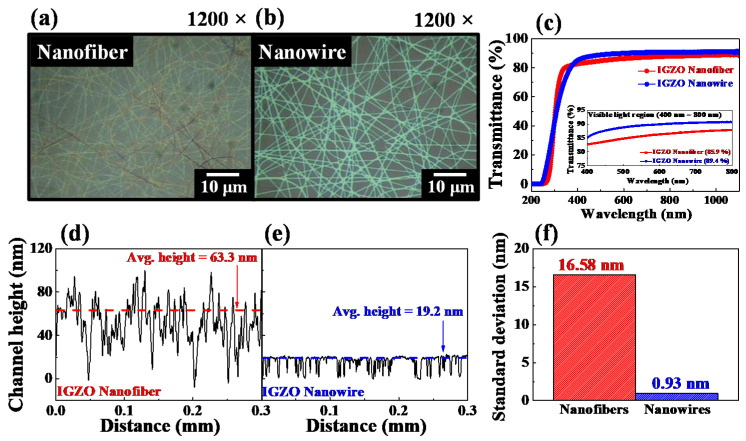
(**a**,**b**) optical microscopy images, (**c**) optical transmittance data (inset shows transmittance data in the visible light region), (**d**,**e**) channel height profiles, and (**f**) standard deviations of the heights of the random networks generated by the electrospun IGZO NFs and the PVP NF template-transferred IGZO NWs.

Figure 3a,b show optical microscopy images (1200× magnification) of the electrospun IGZO NFs and PVP NF template-transferred IGZO NWs, respectively, which indicate that both NFs and NWs had appropriate randomly networked structures.

Figure 3c shows the optical transmittance data of the IGZO NF and NW random networks fabricated on a glass substrate (7059 glass, Corning Inc., Somervile, MA, USA); the data corresponding to the visible light region (400–700 nm) are shown in the inset. The average transmittances of the NF and NW random networks were 85.9% and 89.4%, respectively, indicating that the transmittance of the NFs was lower than that of the NWs. This is because, even in the high-temperature calcination process of the electrospun IGZO NFs, the In, Ga, and Zn components are not completely oxidized, or impurities such as vacancies and polymer matrix still remain [21,22].

Figure 3d,e show the surface height profiles of the IGZO NF and IGZO NW random networks, respectively. The IGZO NFs were randomly laminated during the electrospinning process, resulting in a random network structure with nonuniform thickness and a multilayer channel morphology. The physically stacked multilayer channels of the electrospun IGZO NFs have poor mechanical properties, high contact resistance, and nonuniform electrical properties [23,24,25]. In contrast, a uniform monolayer channel without a randomly stacked structure was formed by the IGZO NWs via PVP NF template transfer. Therefore, the height of the IGZO NW layer was precisely and uniformly adjusted to the deposition thickness of the sputtering system, and the density (surface coverage) of the NW channels was determined by the electrospinning duration of the PVP NFs. Therefore, the proposed method is advantageous in terms of conveniently fabricating FET devices with the required channel morphology because of the independent control of the height and density of the IGZO NWs.

Figure 3f shows the standard deviations of the heights of the NF and NW random networks that were extracted from Figure 3d,e (16.58 nm and 0.93 nm, respectively). The results indicate that the NWs transferred by the PVP NF template had a remarkably uniform thickness compared to the NFs.

**Figure 4:**
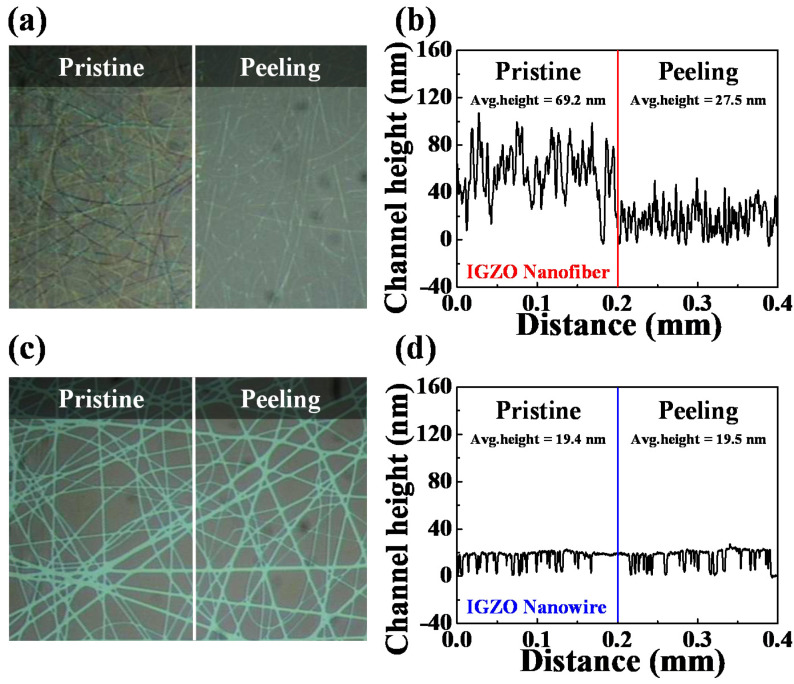
Adhesion tests performed by peeling 3M scotch tape. (**a**,**c**) Optical microscopy images and (**b**,**d**) channel height profiles of random networks of the electrospun IGZO NFs (**a**,**b**) and PVP NF template-transferred IGZO NWs (**c**,**d**).

The mechanical robustness and substrate adhesion properties of the two prepared random-network nanochannels were evaluated using the tape peeling method [26,27,28]. Figure 4 shows optical microscopy images and channel height profiles of the random networks of the electrospun IGZO NFs and PVP NF template-transferred IGZO NWs obtained after the tape-peeling experiments. The results suggest that a significant proportion of the NFs in the electrospun IGZO NF random network were peeled off from the Si/SiO_2_ substrate upon attaching and detaching 3M scotch tape (Figure 4a). In addition, the average channel height significantly decreased from 69.2 to 27.5 nm, indicating inferior robustness and adhesion to the substrate, which was due to the heterogeneous and randomly stacked structures of the IGZO NFs (Figure 3d). In contrast, the PVP template-transferred IGZO NW random network remained intact after the tape-peeling experiment (Figure 4b), and its average height was maintained at ~20 nm. Therefore, the NF template-transferred IGZO NWs exhibited superior transparency, controllability and uniformity of thickness, and excellent mechanical properties, such as robustness and substrate adhesion.

**Figure 5:**
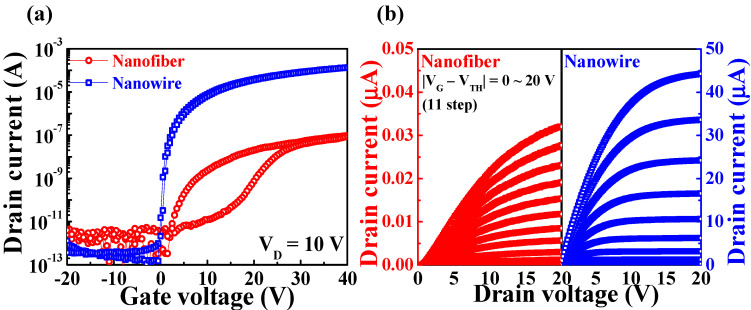
Electrical characteristics of the electrospun IGZO NF FETs and PVP NF-template-transferred IGZO NW FETs: (**a**) transfer curves (*I*_D_–*V*_G_) and (**b**) output curves (*I*_D_–*V*_D_).

Figure 5 shows typical electrical characteristics of the FETs constructed using the electrospun IGZO NFs and PVP template-transferred IGZO NWs. The transfer characteristics (*I*_D_–*V*_G_) of the NF and NW FETs (Figure 5a) were determined using a double-sweep gate voltage from −20 to 40 V at a constant drain voltage (*V*_D_; 10 V), and the output characteristics (*I*_D_–*V*_D_; Figure 5b) were determined by varying *V*_D_ (0–20 V) and |*V*_G_ − *V*_TH_| (0–20 V). The NW FETs were found to exhibit superior electrical performance to that of the NF FETs, with significantly higher on-current, lower leakage current, and a more abrupt change in *I*_D_.

To enable a quantitative comparison of the electrical characteristics of the two types of channels, various electrical parameters (averaged values) were extracted (Table 1). The field-effect mobility (*μ*_FE_) was calculated using Equation (1), where *C*_ox_ is the gate oxide capacitance, *g*_m_ is the transconductance, and *V*_D_ is the drain voltage from the transfer curves [29]. The on/off current ratio (*I*_on_/*I*_off_) and threshold voltage (*V*_TH_) were determined from the transfer curves, and the subthreshold swing (*SS*) was calculated using Equation (2).

(1) $$\mu_{FE}=\frac{L}{WC_{ox}V_{D}}g_{m}$$

(2) $$SS=\frac{d\;V_{G}}{d\log I_{D}}$$

The electrical properties of the PVP NF template-transferred IGZO NW FETs were superior to those of the electrospun IGZO NF FETs owing to their larger *μ*_FE_ (11.81 cm^2^ V^−1^ s^−1^), smaller *SS* (0.32 mV/dec), and higher *I*_on_/*I*_off_ (1.03 × 10^9^). The high *SS* and low *μ*_FE_ of the electrospun IGZO NF FETs lead to high power consumption and poor frequency response [30]. The hysteresis voltage (*V*_H_), which is the difference in threshold voltage between the forward and the reverse gate voltage sweeps in the transfer curves, was determined to be 0.45 V and 12.64 V for the NW and NF FETs, respectively. Clockwise hysteresis occurred in both devices, which can be primarily attributed to electron trapping at the interface states [31,32]. In particular, the significantly greater *SS* and *V*_H_ in the IGZO NF FETs, despite the use of identical gate dielectric materials, indicate the presence of numerous trap states at the dielectric/channel interface or within the channel. To evaluate the uniformity of the electrical performance, the average value and standard deviation (SD) were also calculated using 10 FETs for each channel type. The relative standard deviation (RSD = SD/average) of *μ*_FE_ was 0.29 and 0.38, and that of *SS* was 0.12 and 0.23 for the NW and NF FETs, respectively. Because the value of *V*_TH_ can be zero or negative, the RSD of *V*_TH_ cannot enable a meaningful comparison of uniformity of performance [33]. The SD of *V*_TH_ was 0.13 and 1.31 V for the NW and NF FETs, respectively, with the latter being 10 times greater than the former. The PVP template-transferred IGZO NW FETs had lower deviations for *μ*_FE_, *SS*, and *V*_TH_ than the electrospun IGZO NF FETs, indicating more uniform electrical characteristics in the former. Notably, a significant deviation in the *V*_TH_ of the IGZO NF FETs adversely affects the operational uniformity, which can lead to malfunctioning of the system.

**Figure 6:**
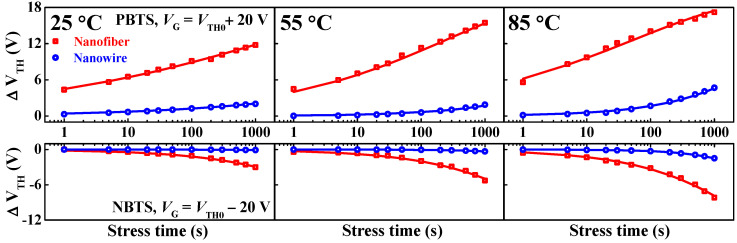
Threshold voltage shifts (∆*V*_TH_) determined by positive-bias temperature stress (PBTS; *V*_G_ = *V*_TH0_ + 20 V) and negative-bias temperature stress (NBTS; *V*_G_ = *V*_TH0_ − 20 V) for the electrospun IGZO NF FETs and PVP NF-template-transferred IGZO NW FETs. The dots represent experimental data, and the solid profiles represent the fitting curves obtained using Equation (3).

Figure 6 shows the time-dependent shifts in the threshold voltage (∆*V*_TH_) of the IGZO NF and NW FETs obtained under positive-bias temperature stress (PBTS) and negative-bias temperature stress (NBTS). These tests are fundamental evaluation methods for determining the stability of FETs based on prolonged gate bias and temperature stressing. For the practical application of the IGZO NFs or NWs, long-term operational stability is crucial for reliable electronic devices. In particular, the evaluation and improvement of device stability are critical for advanced functional materials because of the vulnerability of conventional electrospun NFs to bias stress caused by abundant defects [34]. The PBTS and NBTS conditions were *V*_G_ = *V*_TH0_ ± 20 V and *V*_D_ = 0 V at temperatures of 25, 55, and 85 °C for 10^3^ s, where *V*_TH0_ is the initial threshold voltage of the FETs without gate stress. |∆*V*_TH_|, which is the shift of the threshold voltage, was found to increase with increasing stressing temperature and time. This behavior can be explained by the charge-trapping mechanism in oxygen-related trap states (acceptor-like states) or oxygen vacancy-related trap states (donor-like states) [35,36,37]. When the gate is positively biased, electrons are trapped in the oxygen-related trap states, shifting the threshold voltage in a positive direction. Conversely, a negative gate bias traps holes in the oxygen vacancy-related trap states, shifting the threshold voltage in the negative direction. In addition, because the charge-trapping mechanism is primarily responsible for this shift in threshold voltage, IGZO thin-film transistors (TFTs) with n-type characteristics are generally degraded more severely by the trapping of the majority carriers under the PBTS condition than under the NBTS condition [38]. Moreover, the |∆*V*_TH_| of the NF FETs was considerably higher than that of the NW FETs in both the PBTS and the NBTS tests in the present study. These results indicate the presence of numerous traps at the interface between the gate insulator and the channel and within the channel. In particular, numerous trap states existed in the electrospun IGZO NF channel owing to residual additives. The |∆*V*_TH_| data shown in Figure 6 were adequately fitted using a stretched exponential function, as expressed by Equation (3) [39].

(3) $$∆V_{\mathrm{TH}}=∆V_{0}\left\{1-\exp\left[-{\left(\frac{t}{\tau }\right)}^{\beta }\right]\right\}$$

where ∆*V*_0_ is the ∆*V*_TH_ at infinite stress time, τ is the charge-trapping time constant, and β is the stretched exponent.

**Figure 7:**
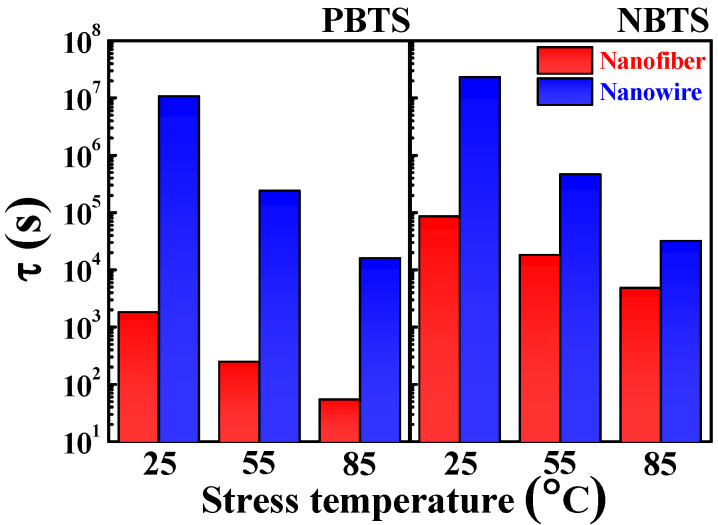
Charge-trapping time constants (τ) of the electrospun IGZO NF FETs and PVP NF-template-transferred IGZO NW FETs determined by the PBTS and NBTS tests. The τ data were extracted by fitting the experimental results to a stretched exponential function.

Figure 7 shows data related to the charge-trapping time constant (τ), which is the time required for a charge to be captured in the trap states during the PBTS and NBTS tests. As the stressing temperature increased, τ decreased independently of the gate bias polarity and channel type. In addition, the IGZO NW FETs had higher values of τ than the IGZO NF FETs in both tests. Notably, the τ of the NF FETs under PBTS was remarkably small, indicating the occurrence of severe degradation by electron traps in the NF FETs. Therefore, the sputtered-film-based IGZO NWs transferred with PVP NF templates enabled the fabrication of FETs with excellent uniform electrical properties and long-term reliability. Consequently, the IGZO NWs designed in this study are anticipated to be effective alternatives to conventional electrospun IGZO NFs.

## 4. Conclusions

Random-network IGZO NWs were fabricated by transferring an electrospun PVP NF template pattern. The PVP NFs, which were used as an etch mask, required a low calcination temperature, and a sputtered IGZO thin film was etched to form randomly networked IGZO NWs. The PVP NF-template-transferred IGZO NWs had a uniform thickness with a monolayer channel, unlike the randomly stacked channels of the electrospun IGZO NFs. The optical transmittance of the IGZO NWs was higher than that of the IGZO NFs because of the low content of impurities such as vacancies and leftover polymer matrix, even at a relatively low process temperature. In addition, the 3M tape-peeling experiment revealed the superior mechanical robustness and substrate adhesion characteristics of the IGZO NWs. FETs were fabricated using the electrospun IGZO NFs and PVP NF-template-transferred IGZO NWs, and their electrical properties, uniformity, and long-term reliability during operation were evaluated. The PVP NF-template-transferred IGZO NW FETs exhibited outstanding electrical characteristics, such as higher *μ*_FE_ (11.81 cm^2^ V^−1^ s^−1^), lower *SS* (0.32 V/dec), larger *I*_on_/*I*_off_ (7.39 × 10^8^), and smaller *V*_H_ (0.45 V), than the electrospun IGZO NF FETs. In addition, the IGZO NW FETs exhibited less deviation in these properties, resulting in excellent operational uniformity. To evaluate the reliability, threshold voltage shifts were determined by applying a prolonged gate bias stress under a temperature stress. In both PBTS and NBTS experiments, the IGZO NW FETs showed a lower ∆*V*_TH_ and a longer charge trapping duration (τ), indicating superior long-term stability. In conclusion, the PVP NF-template-transferred IGZO NWs can presumably be applied in various fields such as flexible electronics, optics, and sensing and replace conventional electrospun IGZO NFs.

## Figures and Tables

**Table 1:** Electrical parameters of the electrospun IGZO NF FETs and PVP NF-template-transferred IGZO NW FETs.

Channel	*μ*_FE_ (cm^2^ V^−1^ s^−1^)		*SS* (V/dec)		*I*_on_/*I*_off_	*V*_TH_ (V)		*V*_H_ (V)
	Avg.	RSD	Avg.	RSD		Avg.	SD	
Nanofibers	0.16	0.38	1.62	0.23	6.28 × 10^4^	9.87	1.31	7.25
Nanowires	11.81	0.29	0.32	0.12	7.39 × 10^8^	0.52	0.13	0.45

## Data Availability

The data presented in this study are available from the corresponding author upon reasonable request.
